# Bright's Disease and Allied Affections of the Kidneys

**Published:** 1886-09

**Authors:** 


### Reviews of Books.

---

*Bright's Disease and Allied Affections of the Kidneys.* By CHARLES W. PURDY, M.D. (Queen's Univ.) London: H. K. LEWIS. 1886.

This work aims at furnishing a systematic, practical, and concise description of the pathology and treatment of the chief organic diseases of the kidneys associated with albuminuria; and it includes a specially full and systematic description of scarlatinal and puerperal nephritis.

After a preliminary chapter on albuminuria, and one on uræmia, the following subjects are treated in order:—Acute nephritis, chronic nephritis, cirrhosis of the kidney, scarlatinal nephritis, puerperal nephritis, lardaceous degeneration, and cyanotic induration of the kidneys. It will be at once seen that the subject is dealt with from the clinical rather than from the pathological point of view, and that the author does not profess to deal with all the diseases of the kidneys.

The much discussed question as to the best tests for albumen in the urine is considered in detail, and the conclusions differ in some respects from those of the Albumen Test Committee of the Clinical Society (*Brit. Med. Journal*, June 5th, 1886). He says (page 44): "Of the five tests for albumin in the urine that have just been considered, the most sensitive are the mercuric and picric; next in order are heat, ferrocyanic, and nitric acid in the cold. The most reliable are the ferrocyanic and nitric acid (Heller's). It may be thought unnecessary to advise the use of so many tests for albumin: some have claimed that one or two are quite sufficient. I know of no test for albumin in the urine which under some circumstances has not misled the observer; and it is therefore my habit, in those rarer cases in which doubt arises, to submit the urine in succession to each of the five tests described." "An impression seems to have gained considerable belief with the profession that the smaller traces of albumin in the urine are of little clinical importance; but it would be difficult to find a more signal mistake in the whole range of clinical medicine, or one behind which more danger to life lurks."

It may be observed that these traces of albumen are of much greater significance in the case of the male sex: the urine of women is so constantly contaminated with mucus and vaginal or uterine products, that little or no reliance can be placed on the traces of albumen found in the urine unless the latter has been removed by means of the catheter. These traces are admittedly of the gravest clinical significance, when found in the urine of the male patient; but no one would attach any significance to this fact in the case of the woman in the absence of some other indications of renal cirrhosis. Our author advises that two tests should always be employed: "These should consist of one of the most sensitive, as the mercuric, and one of the most trustworthy, as the ferrocyanic: in case no reaction be observed with either, it may be safely assumed that no albumin is present: if, on the contrary, a reaction be obtained with one, or both, then the whole five should be systematically gone through with as described." This advice is doubtless good: but, when the rule is systematically adopted of examining the urine of every patient, the amount of time involved will be too great: and further, the fallacies involved in each individual test are few and easily avoided: the busy practitioner will probably find it more convenient to use one test habitually, and reserve the others for those specimens where the first reaction gives an unreliable result. It must be better, as with drugs so with tests, to use those with which one is most familiar and has had most reason to trust: the fallacies soon become recognised, and can be easily avoided, whereas in using so many tests there must be more ambiguity and some conflicting evidence.

One advantage of the picric acid method, giving it a decided pre-eminence over the other tests, is that it may be used in the manner advised by Dr. George Johnson for the estimation of the quantity of albumen present. The method is simple, and appears to be reliable: a solution of five grains of picric acid to the ounce being added to a corresponding bulk of urine, in one of Esbach's tubes,* the amount of precipitated albumen is read off on the scale after standing for twenty-four hours, the figures indicating parts by weight in a thousand per volume, or grammes of dried albumen in a litre. This method would appear to be much simpler and more ready of application than that devised by Dr. Oliver,

* *Lancet*, July 10th, 1886.

in which the standard of opacity of a known solution of albumen, precipitated by the mercuric test, is imitated in ground glass, and kept as a standard with which specimens of urine, after precipitation with the mercuric test, and varying degrees of dilution, may be compared.

The short outline of the hygienic treatment of albuminuria is well worthy of careful perusal, and calls attention to various dietetic points on which there should be little difference of opinion, and almost as little diversity of practice: the limitation of the supply of albumen in the food, and the necessity for a liberal use of non-irritating fluids, are points which are very frequently neglected. The author advises ordinary distilled water, but still better for some patients would be the aerated distilled form sold as "Salutaris water."

"In the treatment of acute nephritis no time should be lost in rendering the urine alkaline, and maintaining it so by the administration of a saline, preferably the acetate or the citrate of potassium. The former should be given in doses of ten grains to half a drachm, and the latter in doses of twenty to sixty grains, both largely diluted with water, and repeated every two to five hours, according to the effect on the urine." As a reason for this practice, the author is in concert with observers in general in showing that renal casts, being mostly of an albuminous nature, are soluble in alkaline solutions, that they soon disappear from alkaline solutions outside of the body, and are rarely met with in alkaline urine. It is, therefore, easy to cause the urine itself to dissolve the plugs in the uriniferous tubes, and so effect their removal; hence the utility of these agents rests on a more solid basis than popular favour.

The use of peptonised milk is mentioned by our author only in order that he may give a caution with regard to it, because of certain reported cases in which its use has induced *albuminuria.* He wisely abstains from giving an authority for this statement, but surely he must be familiar with the fact that peptones in excess are eliminated by the kidneys, and will be precipitated by picric acid: that this precipitate is not albumen may be at once seen by its ready solubility on heating the contents of the test-tube; and further, that it is peptone may be confirmed by the copper and potash test. The presence of a little peptone in the urine would be an indication for diminishing the quantity of peptonised milk, but certainly not for withholding it if the indications for its use were still existing; it is only in those patients who take three or four pints of peptonised milk daily that any such peptone reactions are found, and cases of peptone poisoning are of very rare occurrence. The results of treatment of chronic nephritis by a purely milk diet, as advocated by Dr. George Johnson, have been so encouraging that there is every reason to persevere in this kind of treatment ; and where the coagulable milk may disagree, the peptonised milk will be the best possible substitute.

As regards the value of drugs in chronic nephritis, Dr. Purdy remarks that he has usually found a decrease in the quantity of albumen as a result of the administration of tannate of sodium, and of fuchsin: he also advises chloride of gold to check the growth of new connective tissue in cases of renal cirrhosis. Amongst the drugs not in everyday use he mentions the apocynum cannabinum, the fluid extract of which, in doses of five to twenty minims, he finds to be an efficient diuretic and cardiac stimulant. His experience of ergot is summed up in the following paragraph: "Ergot, also, has been used in the acute grades of this disease, principally with a view to check the discharge of blood from the kidneys. It sometimes effects this purpose, but with much less certainty and efficiency than digitalis."

We have directed action to only a few of the less familiar points described in detail in this volume: they will serve to indicate that the subject is fully and carefully described by a good clinical observer of large experience, who may well be consulted as a reliable guide in the difficult task of treatment of diseases too often considered to be incurable, but for which much may be done by well-timed and judicious interference.

